# A Comprehensive Review of Current Advancements in the Diagnosis, Treatment, and Long-Term Implications of Hypertensive Disorders of Pregnancy

**DOI:** 10.7759/cureus.85025

**Published:** 2025-05-29

**Authors:** Fernanda Ayumi Fukuya, Kiranmayee S Nemalapuri, Afshan Jabeen, Ana Vitoria Moreira de Marchi Apolaro, Hansika Venkatesan, Anjana K Peter, Yu Min Krystal Chen, Rabia Rehman, Ayodele Ifeoluwa Mary, Nahila A Pathan, Ramsha Ali

**Affiliations:** 1 Internal Medicine, University of Mogi das Cruzes (UMC), Mogi das Cruzes, BRA; 2 Medicine, Massachusetts College of Pharmacy and Health Sciences, Boston, USA; 3 Internal Medicine, Ayaan Institute of Medical Sciences, Moinabad, IND; 4 Medicine, Faculdade de Medicina Santa Marcelina, São Paulo, BRA; 5 Internal Medicine, Yerevan State Medical University After Mkhitar Heratsi, Yerevan, ARM; 6 Internal Medicine, Government Medical College, Nagapattinam, IND; 7 Internal Medicine, D. Y. Patil University School of Medicine, Navi Mumbai, IND; 8 Internal Medicine, St. George’s University, West Indies, GRD; 9 Public Health, Glasgow Caledonian University, Glasgow, GBR; 10 Family Medicine, American University of Antigua, Chino, USA; 11 Medicine and Surgery, Peoples University of Medical and Health Sciences For Women, Nawabshah, PAK

**Keywords:** cardiovascular disease, healthy lifestyle, long-term implications, normotensive pregnancies, pulmonary hypertension in pregnancy

## Abstract

This narrative review aims to analyze the existing data on the diagnosis, risk factors, treatment, prevention, and long-term implications of hypertension in pregnancy. An extensive search was conducted using the PubMed database to identify relevant studies from the past 10 years. These studies indicate that hypertension during pregnancy is a leading cause of maternal and infant mortality in the United States. Early identification of high-risk individuals, along with improved prenatal screening and early intervention strategies, has been shown to enhance maternal care. Home blood pressure monitoring (HBPM) is a simple and reliable method for measuring an individual's blood pressure (BP). Hypertensive disorders of pregnancy can be predicted using biomarkers such as alpha-fetoprotein, free beta-human chorionic gonadotropin (hCG), and D-dimer in blood samples. Additionally, the soluble fms-like tyrosine kinase 1-to-placental growth factor (sFLT-1/PLGF) ratio is a key biomarker for diagnosing early- and late-onset preeclampsia. The administration of antihypertensive medications for mild-to-moderate hypertension is beneficial in preventing adverse maternal and fetal outcomes. First-line (such as labetalol) and second-line (including nifedipine, methyldopa, and hydrochlorothiazides) drugs are commonly used. Aspirin may be used prophylactically in cases of chronic hypertension to prevent preeclampsia. In addition to this, planned delivery at 38 weeks of gestation has been associated with a reduction in adverse outcomes for both mother and fetus. Regular BP monitoring, smoking cessation, and adherence to a healthy diet and lifestyle can help reduce the long-term complications of hypertension during pregnancy.

## Introduction and background

Did you know that patients with gestational hypertension or preeclampsia have a 3.5 times increased risk of developing chronic hypertension in the future [1]?

Hypertensive disorders of pregnancy account for around 5%-10% of maternal and fetal complications worldwide [2]. If a hypertensive woman conceives or is known to be taking medications to treat hypertension before pregnancy, this is referred to as chronic hypertension. Gestational hypertension is defined by raised blood pressure (BP) after 20 weeks of gestation without noticeable proteinuria. Similarly, preeclampsia is characterized by an elevation in BP after 20 weeks, accompanied by significant proteinuria or damage to end organs. Chronic hypertension with superimposed preeclampsia means a woman with chronic hypertension who develops preeclampsia. These conditions can lead to severe and fatal complications, renal dysfunction, pulmonary edema, seizure, stroke, cardiovascular effects, preterm birth, stillbirth, small for gestational age (SGA), and neonatal asphyxia [3].

Given these risks, research on preeclampsia prevention is crucial. For example, a recent study demonstrated that aspirin plays a vital role in preventing preeclampsia in high-risk women. However, further research is still needed, as researchers were unable to provide an ideal prophylactic dose of aspirin [4].

Hypertensive disorders of pregnancy continue to present a significant challenge for healthcare providers due to their complex causes, unpredictable course, and long-term effects on both the mother and child [5,6]. This narrative review seeks to influence clinical practice and future research by examining the most relevant and up-to-date data. Furthermore, by identifying gaps in current diagnostic methods, management, and follow-up strategies, this study aims to assist healthcare professionals in implementing interventions to reduce the impact of hypertensive disorders of pregnancy.

According to reports, the prevalence of hypertension during pregnancy is the leading cause of maternal and infant mortality in the United States. This is noted as one of the risk factors for cardiovascular diseases [1]. The researchers summarized that the risk of developing chronic hypertension after gestational hypertension and preeclampsia is higher in White women with greater BMI (>35/kg/m^2^), women with early-onset preeclampsia, and preeclampsia with severe features as compared to normotensive pregnancies [7]. The chances of developing cardiovascular diseases such as coronary artery disease, congestive heart failure, and stroke increase significantly [1]. 

The International Society for the Study of Hypertension in Pregnancy (ISSHP) recommends that ideal management involves regular review of charts and strict BP control, with a target of 110-140/85 mmHg. While mercury sphygmomanometers were historically the gold standard, they are being phased out due to irregular accuracy. Aneroid devices, particularly wall- or trolley-mounted models, are now preferred, with automated monitors under validation for use in pregnancy [8]. Home BP monitoring (HBPM) is also becoming an essential daily component of care. Postpartum management includes indefinite follow-ups with regular BP monitoring and counseling for a healthy lifestyle and diet [9].

Proteinuria is a crucial component in the diagnosis of preeclampsia. The 24-hour urine collection method is susceptible to human error. Therefore, spot protein creatinine ratio (PCR) is being facilitated for efficient management. Unfortunately, 24-hour urine tests and spot PCR are not available in most clinical settings, and many physicians have to rely on urine dipstick assessment. However, we should not rely on the urine dipstick test alone. Even if the dipstick test shows protein +2, it is advisable to start treatment for preeclampsia only if the clinical suspicion is positive [8]. 

This narrative review aims to critically analyze the advancements from the past 10 years regarding diagnostic tools and methods, introduction, and efficacy of new pharmacological and non-pharmacological treatments and highlight the health consequences and outcomes of hypertensive disorders of pregnancy in women throughout their reproductive and early middle years. Finally, we will explore how these advancements can lead to better outcomes for the maternal-fetal binomial in the future.

This study is a narrative review conducted using the PubMed database. After applying the inclusion and exclusion criteria, a total of 65 articles including randomized controlled trials, systematic reviews, and meta-analyses on the diagnosis, treatment, and outcomes of hypertensive disorders of pregnancy were included in this review. The inclusion criteria consisted of studies conducted on human adults within the last 10 years and published in English.

The primary aim of this narrative review is to critically analyze the existing literature on the diagnosis, treatment, and long-term implications of hypertensive disorders of pregnancy, highlighting significant findings, contradictions, limitations, and areas requiring further research.

## Review

**Figure 1:**
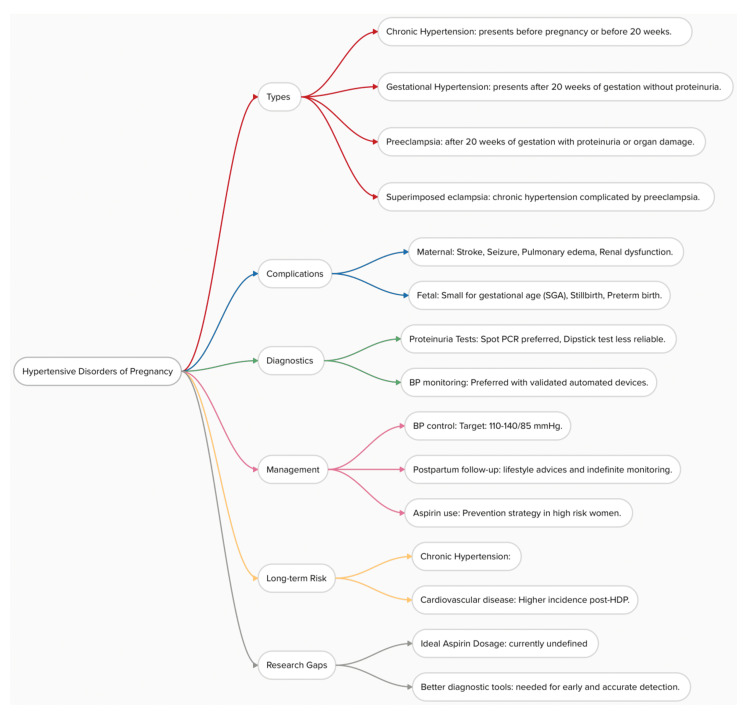
Overview of hypertensive disorders of pregnancy This concept map summarizes the types, complications, diagnosis, management, long-term risks, and research gaps related to hypertensive disorders of pregnancy. Image Credits: Yu Min Chen and Kiranmayee S Nemalapuri.

Figure 1 provides a comprehensive overview of hypertensive disorders of pregnancy. 

Discussion 

Extensive studies have explored the relationship between hypertensive disorders of pregnancy and the increased risk of future cardiovascular complications for both mothers and their children, particularly in terms of prevalence and risk factors. Hypertensive disorders in mothers and fetuses affect approximately 5%-10% of pregnancies and are strongly linked to severe maternal and fetal outcomes. The outcomes include eclampsia, cerebrovascular events, pulmonary edema, severe renal impairment, liver failure, HELLP syndrome, placental abruption, and neonatal complications such as fetal or neonatal death, intraventricular hemorrhage, and hyaline membrane disease [10]. Although there have been significant advancements in the management of these disorders, there remains a gap in understanding how early intervention strategies, such as low-dose aspirin and specific therapies, can further lower BP levels and enhance neonatal and maternal safety. Certain treatments such as labetalol and vitamin E were found to be effective in reducing the BP and protein levels, but further studies are required to evaluate long-term outcomes for both the mother and baby [11].

Our study aligns with previous study findings, confirming the benefit of combination therapies and low-dose aspirin in regulating hypertensive disorders [11]. However, we noted differences in long-term cardiovascular outcomes that have not been thoroughly explored in other studies. For instance, our study extends the scope to include long-term cardiovascular health, which may clarify the contrasting results, while other studies focused mainly on short-term maternal and fetal outcomes [11]. Certain discrepancies may also be attributed to variations in methodology and population characteristics, such as women with a higher pre-pregnancy BMI. Our research highlights the need for long-term cardiovascular health in postpartum women, an aspect that is often overlooked in current studies [12]. 

The study also questions prior assumptions regarding the temporary nature of hypertension during pregnancy by demonstrating ongoing target organ damage and increased risk of vascular disorders [13]. Theoretically, our findings enhance the understanding of hypertensive disorders and their influence on long-term health, particularly in terms of cardiovascular risk. Furthermore, the study highlights the importance of continuous postpartum monitoring, emphasizing the necessity for improved follow-up care to manage elevated risks of chronic hypertension and cardiovascular diseases [1].

An unexpected outcome of our study was the higher prevalence of target organ damage observed within our cohort, suggesting that hypertensive disorders during pregnancy may result in long-term effects on various organ systems. This finding has significant implications for healthcare providers, emphasizing the need for enhanced monitoring and intervention in postpartum to prevent chronic health complications. Additionally, the retrospective design of our study has prevented us from controlling all confounding variables, which may have influenced the results. Future studies should concentrate on large-scale, longitudinal research with more diverse populations to validate our results and assess the long-term effects of hypertensive disorders on maternal cardiovascular health. It would also be beneficial to explore the genetic factors that predispose women to hypertensive disorders of pregnancy and how they influence disease progression and management [14].

Risk factors for hypertensive disorders of pregnancy include family history of hypertension and diabetes, previous diagnosis of hypertension before pregnancy (the risk of occurrence of gestational hypertension and preeclampsia increased with the presence of elevated BP and stage 1 hypertension) [15], previous history of preeclampsia in pregnancy, being overweight, unhealthy eating and alcohol consumption [16], female history of polycystic ovarian syndrome [17], higher plasmic values of hormone TT3 [18], and higher sex hormone-binding globulin values (a potential biomarker for pregnancy complications) [19]. It was also found that specific ophthalmic Doppler parameters are associated with the risk of preeclampsia, as preeclamptic women exhibited differences in three out of six measured values compared to normotensive women. These are time-averaged mean peak velocities (MV), pulsatility index (PI), and end-diastolic velocities (EDVs), which have potential use as predictors of preeclampsia risk [20].

Prevention 

Prevention of pregnancy-induced hypertension includes both non-pharmacological and pharmacological approaches. Non-pharmacological strategies involve healthy eating, physical exercise, and control of body weight [16]. Pharmacologically, metformin use in women diagnosed with gestational diabetes has been shown to reduce the risk of pregnancy-induced hypertension compared to insulin treatment [21]. Magnesium supplementation, on the other hand, was found to be insufficient in preventing adverse outcomes of pregnancy-induced hypertension by multiple studies [22,23]. However, He et al. found that patients with pregnancy-induced hypertension have lower serum concentrations of zinc, calcium, and magnesium than normotensive pregnant patients [24]. On the other hand, low-dose aspirin and calcium supplementation were effective in preventing preeclampsia and reducing consequential preterm birth and postpartum bleeding [25].

Genetics 

Advancements in genetics have played a significant role in the diagnosis and prevention of hypertensive disorders of pregnancy and their adverse outcomes. Hypertension is polygenic, and genetic predisposition to hypertension is a major risk factor for preeclampsia. However, environmental factors, such as BMI, modify the risk of preeclampsia [14]. The authors state that sequence variants associated with preeclampsia in the maternal genome are ZNF831/20q13 and FTO/16q12, MECOM/3q26, FGF5/4q21, and SH2B3/12q24. Vitamin D receptor gene ApaI and BsmI polymorphisms may also be associated with the susceptibility risk of hypertensive disorders of pregnancy [26]. Endothelial nitric oxide synthase gene G894T single-nucleotide polymorphisms were also found to play a significant role in gestational hypertension [27]. Generally, preeclampsia is associated with elevated levels of TNF and IL-6, cytokines produced primarily by macrophages [28]. There are also polymorphic variations in fetal genes, transmitted by both the mother and father, which can be linked to the development of hypertension, especially after 20 weeks of pregnancy [29].

Diagnostic Procedures 

When it comes to discrepancies regarding the process of diagnosing pregnancy-induced hypertension, a systematic review and meta-analysis stated that there are differences in the diagnosis of hypertensive disorders of pregnancy regarding home and medical office measurement of BP. Home measurements were up to four units smaller for systolic BP and three units smaller for diastolic BP than measurements taken with a health professional, which could affect diagnosis and treatment efficacy [30]. However, other studies have found different results, indicating that self-BP measurements are effective and similar to office measurements [31].

Significance of Advancements in Diagnosis

Hypertensive disorders of pregnancy are defined as systolic BP ≥ 140 mmHg and/or diastolic BP ≥ 90 mmHg during pregnancy [32]. Therefore, the diagnosis is made through accurate BP measurement.

HBPM has been proven as a reliable and straightforward method for measuring an individual's BP, offering better reproducibility than 24-hour ambulatory BP monitoring (ABPM). HBPM is user-friendly, easy to learn, and does not require visits to a doctor or a hospital, making it accessible and cost-effective. Additionally, it can help differentiate between white coat and masked hypertension [33]. Similarly, a study by Tran et al. concluded that HBPM is particularly useful for hypertensive disorders of pregnancy, offering benefits such as fewer antenatal visits and timely medication adjustments [30]. However, its broader implementation is limited by a lack of sufficient evidence. Future studies should focus on using validated devices, collecting reference data from healthy pregnant women, and establishing clear diagnostic and treatment guidelines [30].

Preeclampsia is defined by gestational hypertension associated with protein in the urine. Proteinuria is confirmed if urinary protein excretion is ≥300 mg over 24 hours in a urine sample or ≥1+ on qualitative dipstick examination. Alternatively, it could be a protein-to-creatinine ratio ≥ 30 mg/mmol or ≥0.3 mg/dL [16].

However, further diagnostic and prognostic tools are currently under research. A study by Chen et al. highlighted the potential for simultaneous fetal aneuploidy screening and prediction of hypertensive disorders of pregnancy using second-trimester blood samples to measure alpha-fetoprotein, free β-hCG, and D-dimer levels [34]. D-dimer alone was also an effective predictive marker for hypertensive disorders of pregnancy. The combination of D-dimer, alpha-fetoprotein, and free beta-hCG demonstrated higher screening efficiency than the traditional alpha-fetoprotein and free β-hCG model, with a receiver operating characteristic (ROC) > 0.800 for severe preeclampsia, preeclampsia, and gestational hypertension. This research demonstrated clinical significance in identifying women at risk for hypertensive disorders of pregnancy, improving screening sensitivity, and supporting early intervention strategies. Expanding the use of these innovative prenatal screening approaches could enhance maternal care, particularly for high-risk pregnancies [34]. 

Additionally, regarding preeclampsia markers, soluble fms-like tyrosine kinase 1 (sFlt-1) levels can increase by 5-6 times and strongly correlate with the disease's incidence. In contrast, placental growth factor (PLGF) levels gradually increase during pregnancy, showing high sensitivity and specificity in predicting preeclampsia. The sFlt-1/PLGF ratio can serve as a key biomarker for both early-onset (20-33 + 6 weeks) and late-onset (>34 weeks) preeclampsia, with ratios exceeding 85 and 110, respectively, indicating a high-risk pregnancy [35].

The role of fetal genetic variations in the development of gestational hypertension is also under investigation. A recent study suggested that multiple sequence variants, such as the maternally transmitted fetal DLK1 rs10139403, contribute to the genetic predisposition to preeclampsia, each exerting a small effect through BP regulation and potentially other pathways. These factors may act through the maternal genome, the fetal genome, or both [14]. 

Treatment 

When it comes to the treatment of hypertension in pregnancy, NICE guidelines stipulate that the commencement of antihypertensive treatment after birth should happen at 150/100 mmHg. Attar et al. evaluated the possible effects of pharmacological management on both maternal and fetal outcomes of patients with mild-to-moderate hypertension during pregnancy and concluded that antihypertensive treatments resulted in a lower risk of maternal outcomes, including severe hypertension [36]. Findings from the Control of Hypertension in Pregnancy Study (CHIPs) trial by Magee et. al are similar to the above finding [37]. The CHIPs was a multicentric trial that investigated whether a tight (target diastolic BP of 85 mmHg) or less tight (target diastolic BP of 100 mmHg) BP control could yield a long-term outcome.

At the end of the study, they concluded that treatment of mild hypertension led to better outcomes. However, this result cannot be relied upon as the main objective of the CHIPs trial was to compare whether less tight BP or tight BP control could yield better outcomes and most of the participants were already on antihypertensive medications, and no comparison was made between patients who had started antihypertensive and those just starting; therefore, conclusions could not be made on the advantages and disadvantages of less tight and tight BP controls. Initially, in 2015, the Society for Maternal-Fetal Medicine (SMFM) disputed lowering the BP to 140/90 mmHg. But, in 2022, they supported the target BP of 140/90 mmHg in patients with hypertension, as it is more beneficial in reducing the risk of long-term maternal complications [36,37]. 

Medical Management 

Most women who experience high BP during pregnancy have a significantly increased likelihood of developing long-term hypertension. However, there is an inconsistency in the management of high BP postpartum [38]. Different recommendations have been made, but the medication preference has not been stated due to a lack of sufficient data [39]. Mostly, the medication choice is dependent on the regimen used during the antepartum period, but the optimal treatment strategy remains unclear due to limited studies [39].

Recently, the predominantly prescribed antihypertensive medications in pregnancy are labetalol, methyldopa, and nifedipine [40]. However, there is no conclusive evidence on the most commonly used medicines during this period [39]. A review by Cairns et al. found some evidence showing that calcium channel blockers, vasodilators, and beta-blockers reduce BP postpartum [41]. However, there was no conclusion on which group of drugs was more efficient [41]. Alavifard et al. compared hydralazine, nifedipine, and labetalol as initial treatment for severely high BP in pregnant women and found no significant difference in the cesarean section rates or maternal side effects [42]. However, nifedipine showed a marginal advantage [42]. Hup et al. argued that nifedipine is preferred due to its dosing rate of 1-2 times a day, compared to labetalol, which is given three times a day [39]. It should also be considered that nifedipine has been shown to have more side effects than labetalol [39]. A recent study by Irfan et al. concluded that most antihypertensive medications are effective; therefore, this lack of restriction allows prescriptions to be tailored to patients' individual needs [43].

Although Hup et al. investigated antihypertensive medication with the optimum effect in the postpartum period and concluded that adding furosemide had a positive impact on women with pulmonary embolism, they proposed a further study using data from health records [39]. Aside from the use of antihypertensives, a study by Chen and Sun, which aimed to evaluate the role of low-dose aspirin combined with calcium supplements for preventing preeclampsia, found that in comparison with the other routine regimens, combining low-dose aspirin with calcium significantly reduced the incidence of preeclampsia, gestational hypertension, and postpartum hemorrhage [25]. However, a limitation of this study was that out of the seven included studies, only one was conducted outside of China, thereby introducing geographical bias [25]. 

Another study also concluded that combining labetalol, low-dose aspirin, vitamin E, and calcium had a high effect on reducing BP and 24-hour urine protein [11]. They also recorded a significant increase in the levels of microRNA-126 and PLGF. The role of microRNA is to promote angiogenesis and maintain vascular endothelial cell homeostasis, thereby acting as a compensatory mechanism in patients with hypertension [11]. Lastly, commencing calcium supplementation before and during pregnancy is of great benefit in reducing the risk of adverse effects such as preeclampsia or pregnancy loss [44]. Magee et al. studied the best time to induce labor in pregnant women with chronic or gestational hypertension [12]. They concluded that planned birth at 38 weeks, compared to usual care, had no difference in poor maternal outcomes [12].

Non-medical Management 

Wen and Liu examined the effects of information-knowledge-attitude-practice (IKAP) and health education combined with cluster-based care in patients with gestational hypertension [45]. Their focus was on providing individual and group health information by distributing health education manuals to hypertensive patients during pregnancy and also to their families. Their study showed that the combination of IKAP and cluster care had a significant difference in preventing maternal outcomes as compared to cluster care alone, with results of more adequate weight and BP regulation, a smaller incidence of negative pregnancy and neonatal outcomes (including placenta previa, cesarean deliveries, unhealthy amounts of amniotic fluid, fetal suffering, and postpartum bleeding), and better postpartum recovery and general life quality (including increased physical exercise, better emotional and mental health, reduced pain and discomfort, and better sleep) [45].

The aim of the treatment of hypertensive disorders of pregnancy is to reduce maternal risks, including cerebrovascular injuries, cardiovascular stress, and renal trauma, and to mitigate fetal risks due to ongoing adverse effects of the antihypertensive medications, which causes a reduction in uteroplacental perfusion [46]. This is of utmost importance, as the aim outlines a dual-protection approach for the mother and the fetus. The initial plan of action begins with adequate prenatal care, timely diagnosis, and efficient delivery of the newborn [47], indicating that prompt diagnosis, regular BP surveillance, and target BP control lead to reduced prenatal risk and severe maternal hypertension. However, cases of preeclampsia prompt caution due to the severity of complications, including hypertension, seizures, and intracerebral hemorrhage [8].

Low-sodium diets remain controversial, suggesting that management plans are tailored according to an individual patient [46]. The first-line antihypertensive medications recommended are oral labetalol, and extended-release nifedipine, methyldopa, and hydrochlorothiazide as second-line treatments [48], providing crucial information on ongoing and acceptable medications for a targeted population. However, the highly recommended medication during chronic hypertension in pregnancy is the daily dose of aspirin 81 mg used prophylactically starting from 12 weeks to 28 weeks of pregnancy, implicating its preventive measures for preeclampsia. In patients with mild gestational hypertension or preeclampsia without severe features, delivery is typically recommended at 37 weeks of gestation or later. However, the mode of delivery depends on factors such as fetal gestational age, fetal presentation, and maternal conditions [8]. According to research, it is best to prolong pregnancy when the mother is <34 weeks; however, in situations where the maternal risk increases with the prolonged pregnancy, it is advisable to take the necessary steps to deliver the fetus. During delivery, corticosteroids are used to promote lung maturity in the fetus. Women may also receive magnesium sulfate, which is associated with a reduced risk of maternal death and fetal benefits [8].

Outcomes 

Rossiter et al. investigated whether and how women could adopt healthy behaviors after a pregnancy complicated by high BP [5]. They found that there is an intense need for structured support to help women adopt healthy lifestyles, especially during the challenges of new motherhood [5]. For example, daily self-weighing has shown a positive impact in postpartum women who underwent pregnancy-induced high BP. Women who weighed themselves more than half of the follow-up days lost more weight than those who did not [49]. Also, the prospective, randomized controlled clinical study by Chen et al. significantly proved that the combination of aerobic and resistance exercises is the most beneficial for long-term health outcomes in patients with hypertensive disorders of pregnancy [35]. Lastly, a randomized controlled trial by Karthiga et al. found that, after 20 weeks of yoga intervention, participants in the yoga group had a lower incidence of hypertension compared to those in the control group [50]. Additionally, lowered cardiometabolic risk, decreased BP, and less inflammation were noticed [50].

According to Jin et al. [51] and Moes et al. [52], high BP before or early in the pregnancy, as well as the intensity of pressure increase, significantly increased the odds of developing hypertensive disorders of pregnancy, as well as of adverse maternal and fetal outcomes, especially regarding increased risk of gestational diabetes, preeclampsia, preterm birth, stillbirth, and also the need for neonatal ICU. This highlights the importance of antenatal BP vigilance for women with abnormal values before or during early pregnancy, to enable early identification and prevention of maternal and fetal risks [51,52]. Accordingly, Moes et al. concluded that an increase in BP in the first trimester, whether close to or above the pressure limits, increases the risk of preeclampsia and fetal growth restriction [52]. Elevated BP remains a problem even after 39 weeks of gestation, according to the secondary analysis of a randomized trial by Bartal et al. [53]. First-time pregnant women who developed high BP at or after 39 weeks (14%) experienced worse placental, cardiorespiratory, hemorrhagic, infectious, and thrombotic outcomes (84%) than those who remained normotensive, with severe cases occurring in approximately 1% of them. They were also more inclined to need a cesarean delivery and postpartum ICU. Peripartum organ damage patterns may help identify women at high risk of future disease, and trials are required to assess interventions that could improve clinical outcomes [53]. High BP during pregnancy is a risk factor for preeclampsia, and when it comes to women who develop preeclampsia, long-term outcomes are even worse [15]. The meta-analysis by Dall'Asta et al. has demonstrated that both early- and late-onset preeclampsia increase the risk of maternal metabolic and cardiovascular complications (major cardiovascular events, chronic hypertension, dyslipidemia, type 2 diabetes, abnormal renal function, and metabolic syndrome) and mortality later in life, while this risk was significantly improved in cases of early-onset preeclampsia [54]. The randomized controlled trial by Magee et al. proved that hypertension in pregnancy was associated with birth weight < 10th percentile, preeclampsia, preterm delivery, elevated liver enzymes, platelets < 100 × 10^9^/L, and prolonged hospital stay [55].

Additionally, it was found that higher BP and heart rate variabilities, along with severe hypertension, are risk factors for adverse maternal and perinatal outcomes, independent of preeclampsia co-occurrence [56]. Yu et al. stated that children exposed to hypertension in utero have developed higher systolic and diastolic BP values than those born to normotensive mothers [32]. However, long-term metabolic alterations in the teenage and adult years were not identified [57].

**Figure 2:**
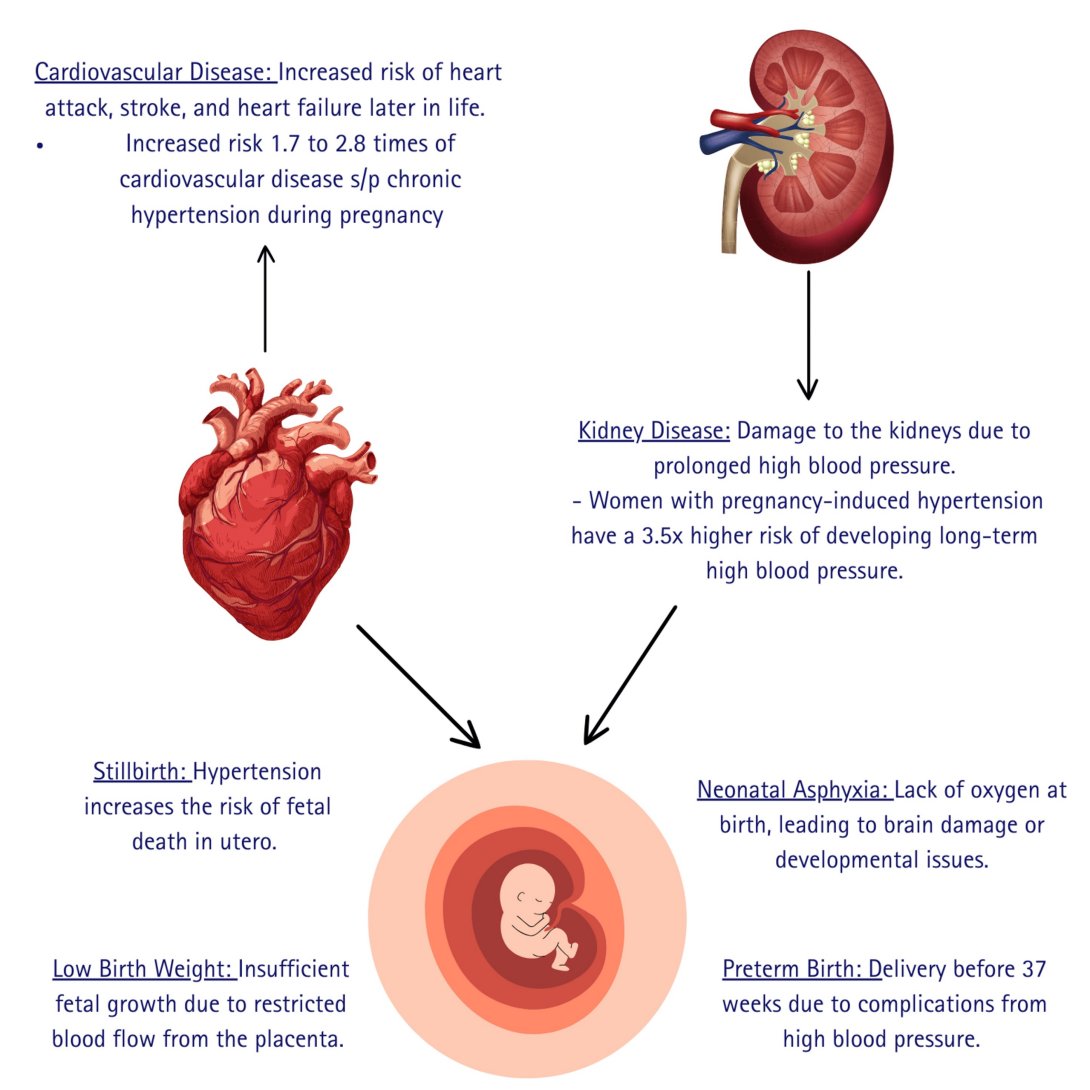
Systemic impact of hypertensive disorders of pregnancy The diagram highlights maternal (cardiovascular and kidney disease) and fetal (stillbirth, low birth weight, neonatal asphyxia, preterm birth) complications resulting from hypertensive disorders during pregnancy. Image Credits: Kiranmayee S Nemalapuri.

Figure 2 shows the systemic impacts of hypertensive disorders of pregnancy.

The systematic review by Barrett et al. established that a history of hypertensive disorders of pregnancy, among other pregnancy-related metabolic conditions, is associated with a higher risk of long-term kidney disease [58]. The risk of end-stage kidney disease (ESKD) was highest among women with a history of preeclampsia [58]. Also, Sukmanee et al. indicated that women who suffer from pregnancy-induced hypertension have an increased risk for future and long-lasting non-gestational hypertension, as well as ischemic heart disease and heart failure later in life, compared with those who had normotensive pregnancies [59]. The first five years after delivery were the most critical when it comes to the risk of perpetuating hypertension [59]. Carefully ongoing BP monitoring and counseling regarding lifestyle modification, therefore, play a pivotal role in preventing the development of persistent hypertension in women with prior episodes of eclampsia and preeclampsia [60]. The systematic review by Cutler et al. agrees that women who were victims of pregnancy-induced hypertension already show evidence of target organ damage, particularly affecting the brain, eyes, heart, kidneys, arteries, and liver, despite early postpartum reductions in BP [13]. Although there is some partial resolution of these target organ changes during the postpartum period, key differences persist, both during the early and late postpartum periods [13,61]. 

Delivery and Postpartum Blood Pressure Control 

When it comes to the delivery outcomes of patients with pregnancy-induced hypertension, Liu et al. investigated the effectiveness of Bakri balloon tamponade (BBT) combined with different suture methods in preventing postpartum hemorrhage in women undergoing cesarean delivery [62]. The intraoperative blood loss, postoperative blood loss at two hours, postoperative blood loss at 24 hours, and decrease in red blood cells and hemoglobin in the BBT combined with modified Hayman's suture group were significantly lower than those in the BBT combined with B-Lynch uterine compression sutures group. After surgery, the levels of inflammation, coagulation function, and sex hormones in both groups improved compared to their pre-surgery levels, with the first group showing a more significant improvement than the second. In addition, the incidence of postoperative adverse events in the BBT + modified Hayman study group was significantly lower than that in the second group [62]. When it comes to BP control after delivery, Cairns et al. found that self-management of postpartum BP yields good results, including improved control, reduced anxiety, and increased adherence to medication [63]. Women found it flexible, practical, and comfortable, particularly with telemonitoring for medication adjustments, which also led to more targeted down-titration of medications and reduction in BP [63]. Muijsers et al. agree and add that self-measurement of BP can be effective for following up women with an increased risk of cardiovascular disease due to previous preeclampsia or HELLP syndrome, compared to those evaluated at the physician's office [33]. According to the meta-analysis by van Oostwaard et al., recurrence rates of hypertension in future pregnancies are relatively low, and the course of disease is milder in recurrent cases [64].

**Figure 3:**
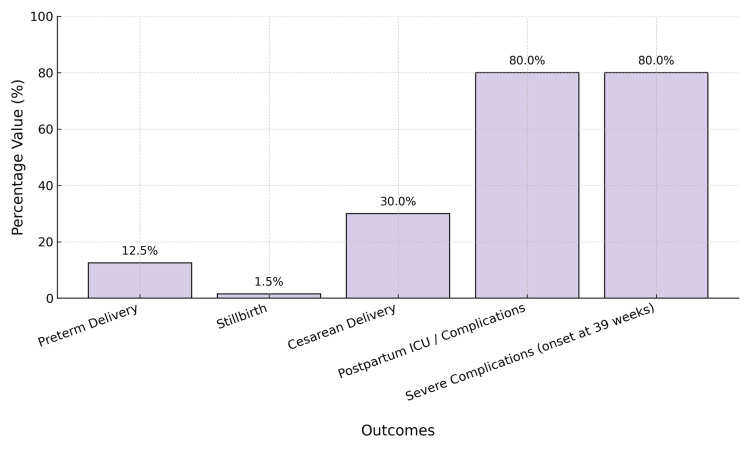
Adverse outcomes of late-onset hypertension in pregnancy This graph shows the key complications, including postpartum ICU care, severe maternal complications, cesarean delivery, preterm birth, and stillbirth, associated with hypertension onset at 39 weeks. Image Credits: Kiranmayee S Nemalapuri.

Figure 3 presents the adverse outcomes of late-onset hypertension in pregnancy. 

Impact of Advancements on Prognosis and Long-Term Outcomes

Significant advancements have been made in the topic of prognosis and long-term management of hypertension during pregnancy. The implementation of pharmacological interventions has notably influenced the prognosis of hypertensive disorders, positively impacting maternal and fetal outcomes. A meta-analysis investigating the administration of antihypertensive medications for mild-to-moderate hypertension in pregnancy demonstrated a marked reduction in the incidence of severe hypertension, preeclampsia, and placental abruption. It did not contribute to adverse long-term outcomes such as SGA infants or fetal mortality [36]. Early detection and proactive management of hypertension are pivotal to improving long-term prognosis. Additionally, another meta-analysis included several randomized controlled trials that revealed that the combination of low-dose aspirin and calcium supplementation significantly mitigated the long-term risks of preterm birth and postpartum hemorrhage by preventing preeclampsia [25]. These findings highlight the vital role of early detection and timely intervention in the prevention of hypertension and preeclampsia. This is just an example [26].

Limitations of the study

The comprehensive nature of the literature review, along with the inclusion of published evidence-based studies, is the strength of this review. Our review was limited by the English language and US population and included articles within the past 10 years. Another limitation is the discretion in choosing the articles that helped extend our knowledge about this topic, leading to selection bias. There are multiple barriers to monitoring the prevalence of hypertension in pregnancy. The use of various EMR systems with a slightly different definition of hypertension can lead to information bias. Another limitation is the short duration of outcomes, as some clinical trials are still ongoing. Some studies collected data from participants at home using BP monitors that differed from those used in other studies. There could also be financial constraints for some studies, which can lead to a lower recruitment rate and funding bias. The sample size can also lead to bias, as a smaller sample size results in less statistical power. Various studies were used in this review, and most of them had their own design. Each clinical trial has its own set of inclusion and exclusion criteria, which can lead to data discrepancies.

**Figure 4:**
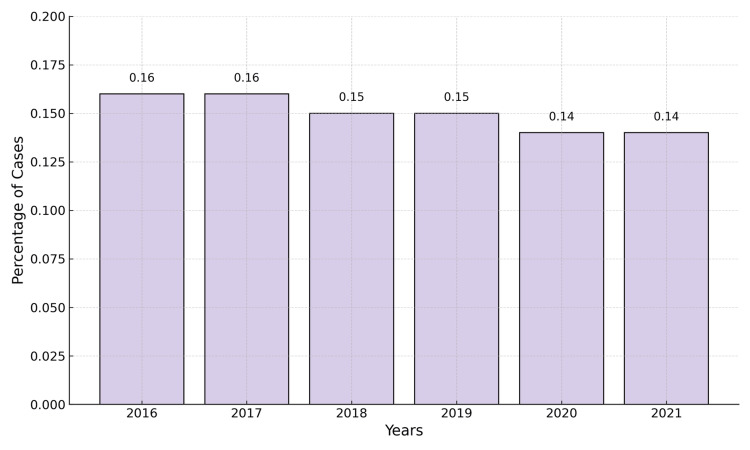
Global percentage incidence of maternal hypertension disorders among females aged 20+ years from 2016 to 2021 Image Credits: Hansika Venkatesan.

Figure 4 shows the global percentage incidence of maternal hypertension disorders among women aged 20+ years from 2016 to 2021.

Summary

Hypertensive disorders of pregnancy complicate approximately 5%-10% of pregnancies and are directly associated with adverse outcomes for both the fetus and the mother [52]. Maternal complications include eclampsia, cerebrovascular events, and placental abruption, while perinatal complications include fetal or neonatal death [10]. Recent advancements in treatment include combination therapy with labetalol, low-dose aspirin, vitamin E, and calcium, which significantly lowered BP and 24-hour proteinuria levels [11]. Another study concluded that oral nifedipine was superior to IV labetalol and IV hydralazine for treating severe hypertension in pregnancy [11]. However, further network meta-analyses are needed to compare drugs within the same class, dosage variations, and administration timing to determine the most effective regimen for preventing hypertensive disorders of pregnancy [6]. The use of aspirin as the first-line treatment for preeclampsia prevention is supported by the need for anticoagulant therapy to treat maternal coagulation dysfunction. Its effectiveness alone is limited, particularly in high-risk cases.

Therefore, the World Health Organization recommends calcium supplementation, especially for pregnant women with low calcium levels or inadequate dietary intake. Although further research is needed, studies suggest that combining low-dose aspirin with calcium supplementation may reduce the incidence of preeclampsia and improve maternal and fetal outcomes [25]. Regarding delivery timing, the When to Induce Labour to Limit Risk in Pregnancy Hypertension (WILL) randomized trial findings support planned birth at 38 + 0-3 weeks as a safe clinical option, with no significant differences in adverse maternal or neonatal outcomes [12]. After childbirth, women who had hypertensive disorders of pregnancy are at a 3.5 times higher risk of developing chronic hypertension and have a 1.7-2.8 times greater risk of cardiovascular disease compared to those without such conditions [1]. These patients might present target organ damage across multiple systems, such as cardiac and renal, persisting to some extent for up to four decades postpartum. The high prevalence highlights the need for further research into disease progression and risk assessment, as well as exploring interventions to improve clinical outcomes [13].

Additionally, following hypertensive disorders of pregnancy, adequate postpartum care is needed, which includes regular BP monitoring, physical activity, smoke cessation, a balanced diet, and weight management to support long-term cardiovascular health [59]. Considering the complexity of hypertensive disorders of pregnancy, this review aims to critically analyze how recent medical research influences the diagnosis and management of these conditions. We seek to raise awareness of potential long-term complications, improve clinical outcomes, and identify knowledge gaps that could guide future research.

## Conclusions

After the critical analysis of several works of literature, we were able to find compelling evidence that the risk factors include a family history of hypertension, diabetes, and a previous history of preeclampsia in pregnancy. High plasma values of the hormone TT3 and sex hormone-binding globulin were found to be potential biomarkers for detecting pregnancy complications. As far as diagnosis, spot PCR is effective in early detection and management, but its availability in most clinical settings is limited. Regular BP monitoring and daily HBPM are found to be effective. Regarding the therapeutic approach, a combination of low-dose aspirin, labetalol, vitamin E, and calcium seems to lower BP. Oral nifedipine helps in treating severe hypertension, while calcium helps in preventing gestational hypertension in nulliparous/primigravida women. Aspirin is given prophylactically for chronic hypertension. A target BP of 140/90 mmHg is more beneficial in reducing the risk of long-term maternal complications. Treatment of mild hypertension leads to better outcomes. Aerobic and resistance exercises were found to be effective. Planned birth at 38 weeks significantly improved maternal and fetal outcomes. Postpartum follow-ups are needed for regular BP monitoring along with counseling for a healthy lifestyle and diet to reduce long-term complications. However, larger studies are needed to confirm these findings and their underlying mechanisms. Hence, we conclude that there is an urgent need to create awareness between healthcare providers and expectant mothers to help with early detection, appropriate management, and prevention of hypertensive disorders. Despite progress in diagnosing and managing hypertensive disorders of pregnancy, they still manage to be a significant concern for both mother and fetal health worldwide, suggesting further research on understanding their development, genetic involvement, screening techniques, novel therapeutic approaches, and long-term implications to have better consequences for both mothers and their infants.
